# Preparation and Characterization of Cellulose Composite Hydrogels From Tea Residue and Single-Walled Carbon Nanotube Oxides and Its Potential Applications

**DOI:** 10.3389/fchem.2021.651566

**Published:** 2021-05-04

**Authors:** Zhijun Liu, Dianxin Li

**Affiliations:** Guangdong Polytechnic of Science and Trade, Guangzhou, China

**Keywords:** tea residue, cellulose, ionic liquid, hydrogel, single-walled carbon nanotube oxide

## Abstract

Hydrogels were prepared from tea cellulose with the addition of single-walled carbon nanotube oxides in 1-allyl-3-methylimidazolium chloride. Single-walled carbon nanotube oxides/tea cellulose hydrogels (TCH-SWNTs) were characterized by Fourier transform infrared, x-ray diffraction, texture profile analysis, and thermogravimetric analysis. The adsorption capacity of methylene blue using the prepared hydrogels was also investigated. The hydrogels exhibited greater thermal stability and intensive textural property with the addition of single-walled carbon nanotube oxides. Compared with undoped TCHs, the weight loss peak moved from 280 to 323°C, and the values of hardness, fracturability, gumminess, and resilience were 8.4, 5.3, 10.8, and 1.9, respectively, times higher than that of TCHs. As an absorbent of methylene blue, TCH-SWNTs accorded to a pseudo-second-order kinetic model, good adsorption capacity (13.8 mg/g), and good adsorption ratio (27.59%) and showed potential as a drug carrier.

## Introduction

The production and consumption of tea is estimated to increase in the next decade to satisfy the increase in demand for tea in developing countries, e.g., India and China. In 2016, tea production all over the world increased and exceeded 5.7 million tons. Every year, a large amount of tea residue is produced after consumption of various kinds of tea. It is an interesting and urgent issue to utilize low-value tea residue. Recently, cellulose abstracted from the waste of various fruits and vegetables, e.g., pineapple peel and bamboo shoot, has been used to prepare composite hydrogels (Dai and Huang, 2016, 2017a; Liu and Huang, 2016). Cellulose, the major component of tea residue, is a linear chain polymer composed of β-D-glucopyranose units and linked by β-1,4-glycosidic bonds and contains massive intra- and inter-molecular hydrogen bonding (Klemm et al., 2005). Cellulose has advantages of low cost and of being non-toxic, renewable, biocompatible, modifiable, and biodegradable, but it is too insoluble in most solvents to be applied in an industry because of intra-molecular and inter-molecular hydrogen bonds (Dai and Huang, 2017b). Since ionic liquids were found to be efficient solvents for cellulose at room temperature (Rogers and Seddon, 2003), a lot of them, e.g., 1-allyl-3-methylimidazolium chloride, 1-butyl-3-methylimidazolium chloride, and 1-ethyl-3-methylimidazolium chloride, were applied to process cellulose and prepare cellulose hydrogels (Wu et al., 2004; Hu et al., 2013). Cellulose-based hydrogels are suitable for embedding and immobilizing some functional components due to their net structure (Bardi and Koutinas, 1994; Richins et al., 2000; Francotte and Huynh, 2002). Single-walled carbon nanotubes are graphite–carbon isomers with more complex structures (Rebelo et al., 2016) and related properties for diverse applications. Because of high surface areas and special structures, single-walled carbon nanotubes can enhance solubilization and delivery of curcumin (Yuan et al., 2016), efficiently eliminate toxic phenol (Dehghani et al., 2016), sensitively detect aflatoxin B1 (Zhang et al., 2016), and so on.

In this study, we prepared composite hydrogels with single-walled carbon nanotube oxides (SWNTs) and tea residue cellulose mixed in 1-allyl-3-methylimidazolium chloride. The single-walled carbon nanotube oxides/tea cellulose hydrogels (TCH-SWNTs) were characterized by Fourier transform infrared (FTIR), x-ray diffraction (XRD), texture profile analysis (TPA), and thermogravimetric analysis (TGA). Methylene blue was used as a drug molecule model to investigate the adsorption properties, e.g., capacity, ratio, and kinetics, of TCH-SWNTs.

## Materials and Methods

### Materials and Reagents

Oolong tea leaf was obtained from South China Agricultural University. Single-walled carbon nanotube oxide (SWNTs) was purchased from Guangzhou Feibo Co., Ltd., Guangzhou, China. We purchased 1-allyl-3-methylimidazolium chloride [[AMim]Cl] from Shanghai Chengjie Chemical Co., Ltd., Shanghai, China. Methylene blue was purchased from Shanghai Chemical Co., Ltd., Shanghai, China.

All other chemical reagents used in this study were of analytical grade.

### Tea Cellulose Extraction

Tea cellulose was extracted from tea residue according to the method of Hu (Hu et al., 2010). Oolong tea leaves were immersed in 90°C deionized water for 30 min, and this process was repeated three times to simulate tea beverage processing. After filtration and drying at 60°C, the tea residue was ground to powder and sifted through a 60-mesh sieve. The obtained tea residue powder was processed by the following four steps: first, degreasing with petroleum ether at a ratio of 1:10 (g/ml) for 2 h; second, depigmentation with acetone at a ratio of 1:10 (g/ml) for 2 h; third, delignification with a pH 4 sodium chlorite solution at a ratio of 1:20 (g/ml) at 75°C for 2 h;. and finally, removal of semi-cellulose with a potassium hydroxide solution (10%, w/v) at a ratio of 1:20 (g/ml) for 10 h. After being washed in turns with deionized water and ethanol (95%, v/v) until the filtrate became neutral, the tea cellulose was dried at 60°C and became available for the preparation of hydrogels.

### Hydrogels Preparation

**Figure 1:**
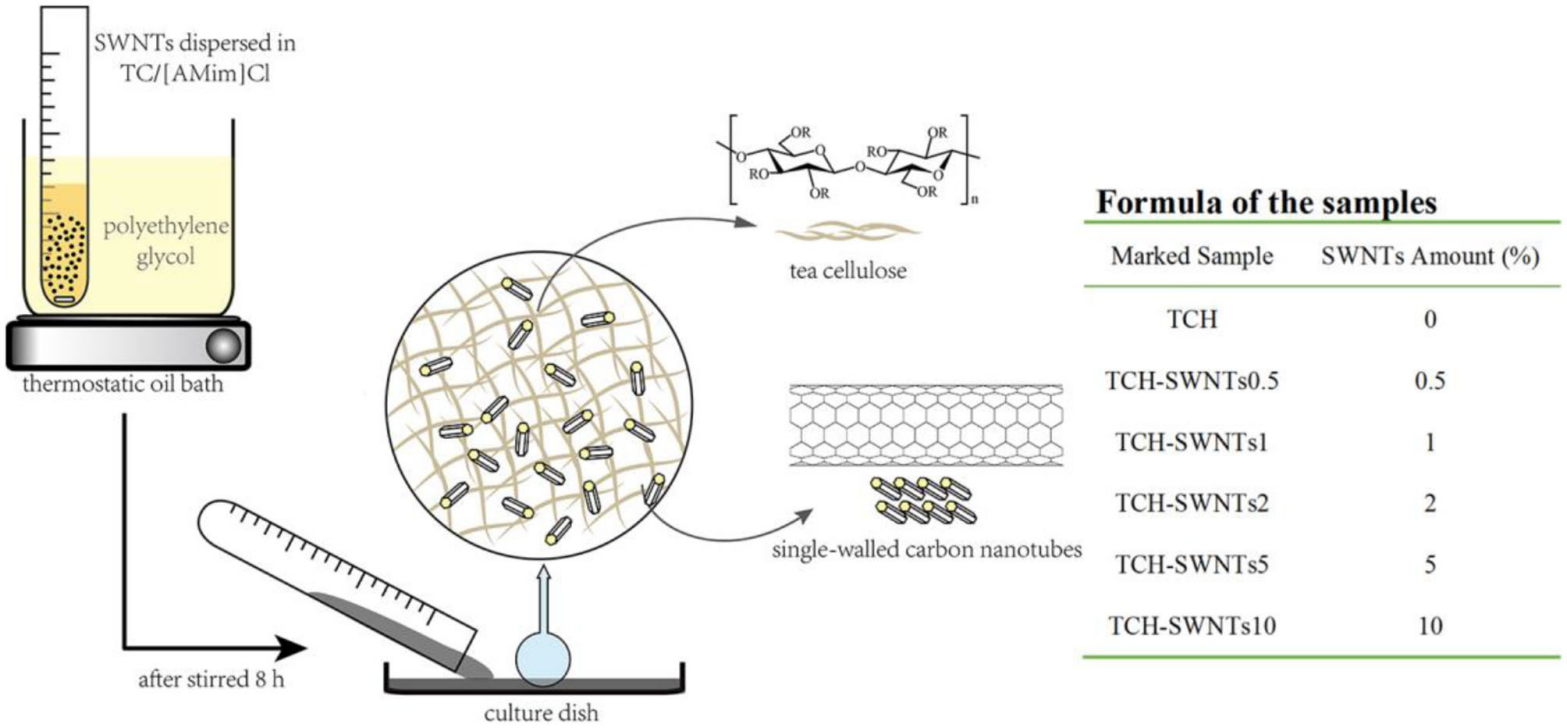
The preparation process and formula of TCH-SWNTs.

The preparation process of the hydrogels is shown in Figure 1. TCH-SWNTs were prepared according to the method of Kadokawa (Kadokawa et al., 2008, 2009). The tea cellulose (0.5 g) and [AMim]Cl (10 g) were mixed in a test tube and stirred at 100°C for 5 h. In accordance with the formula of the samples, as shown in Figure 1, a certain amount of SWNTs was added and stirred at 100°C for 5 h until they fully dispersed. Subsequently, the viscous mixture in the test tube was poured into a petri dish to spread out. The resulting mixture was slowly immersed into deionized water to form composite hydrogel after cooling down to room temperature. TCH-SWNTs were dried using a freeze dryer for subsequent characterization analysis and adsorption experiments. The TCH without SWNTs (TCH) was processed the same way and was used as the comparison sample.

### Characterization

The FTIR spectra were recorded using a Fourier-transform infrared spectroscope (VERTEX-33, Bruker, Berlin, Germany) from 4,000 to 400 cm^−1^ at a resolution of 2 cm^−1^ (Lin et al., 2016). The XRD patterns were measured using an x-ray diffractometer (D8 ADVANCE, Bruker, Berlin, Germany) in the region of the diffraction angle (2θ) from 4 to 60°, in which the step length of scanning was 0.04°, and the scanning velocity was 38.4 s/step. Cu Kα (λ = 1.5406 Å) was used at 30 mA 40 kV. TGA was performed and recorded using a thermobalance instrument (TGA-Q500, TA Instruments, New Castle, DE, USA) from 30 to 600°C at a heating rate of 20°C/min in a N_2_ atmosphere. The texture profiles of the hydrogels, e.g., cohesiveness, fracturability, gumminess, hardness, resilience, and springiness, were characterized using a texture analyzer (TA-XT2i, Stable Microsystems, Surrey, UK) according to the method of Hu (Hu et al., 2010). After being sheared to adiameter of 2.5 cm and a thickness of 0.8 cm, the hydrogel sample was subjected to two-cycle compression to 30% of the initial thickness at 5 mm/s pre-speed, 1 mm/s cross-head speed, and 5 mm/s post-speed.

### Adsorption Experiments

As the drug model, methylene blue was used to investigate the adsorption capacity and adsorption rate of the hydrogels. TCH-SWNT (0.05 g) was immersed in the solution of methylene blue (100 mg/L, 25 ml). The absorption value (665 nm) of the methylene blue solution was recorded using a spectrophotometer (UV-1800, Shimadzu, Tokyo, Japan) at a given time. The remaining methylene blue content in the solution was estimated using the regression equation (*y* = 0.232*x*−0.029) derived from the concentration of methylene blue standard solution (1, 2, 3, 4, and 5 mg/L) and its absorption value.

Adsorption rate (*R*_*e*_) and adsorption capacity (*Q*_*e*_) were calculated according to the following equations (Kannan and Sundaram, 2001; Hameed et al., 2007):

(1) $$\begin{array}{l} Q_{c}=\frac{c_{o}-c_{t}}{m}\times v \end{array}$$

(2) $$\begin{array}{l} R_{c}=\left(1-\frac{c_{t}}{c_{o}}\right)\times 100\% \end{array}$$

where *c*_0_ and *c*_*t*_ are the methylene blue concentrations at the initial time and a given time, respectively, *m* is the weight of the prepared composite hydrogel sample, and *V* is the volume of the methylene blue solution.

### Statistical Analysis

Data statistics and significance analysis were processed using Minitab (Version 17.1.0, Minitab Inc., State College, PA, USA). The probability value *p* < 0.05 represented significant difference, and the probability value *p* < 0.01 represented extremely significant difference.

## Results and Discussion

### FTIR and XRD Analysis

**Figure 2:**
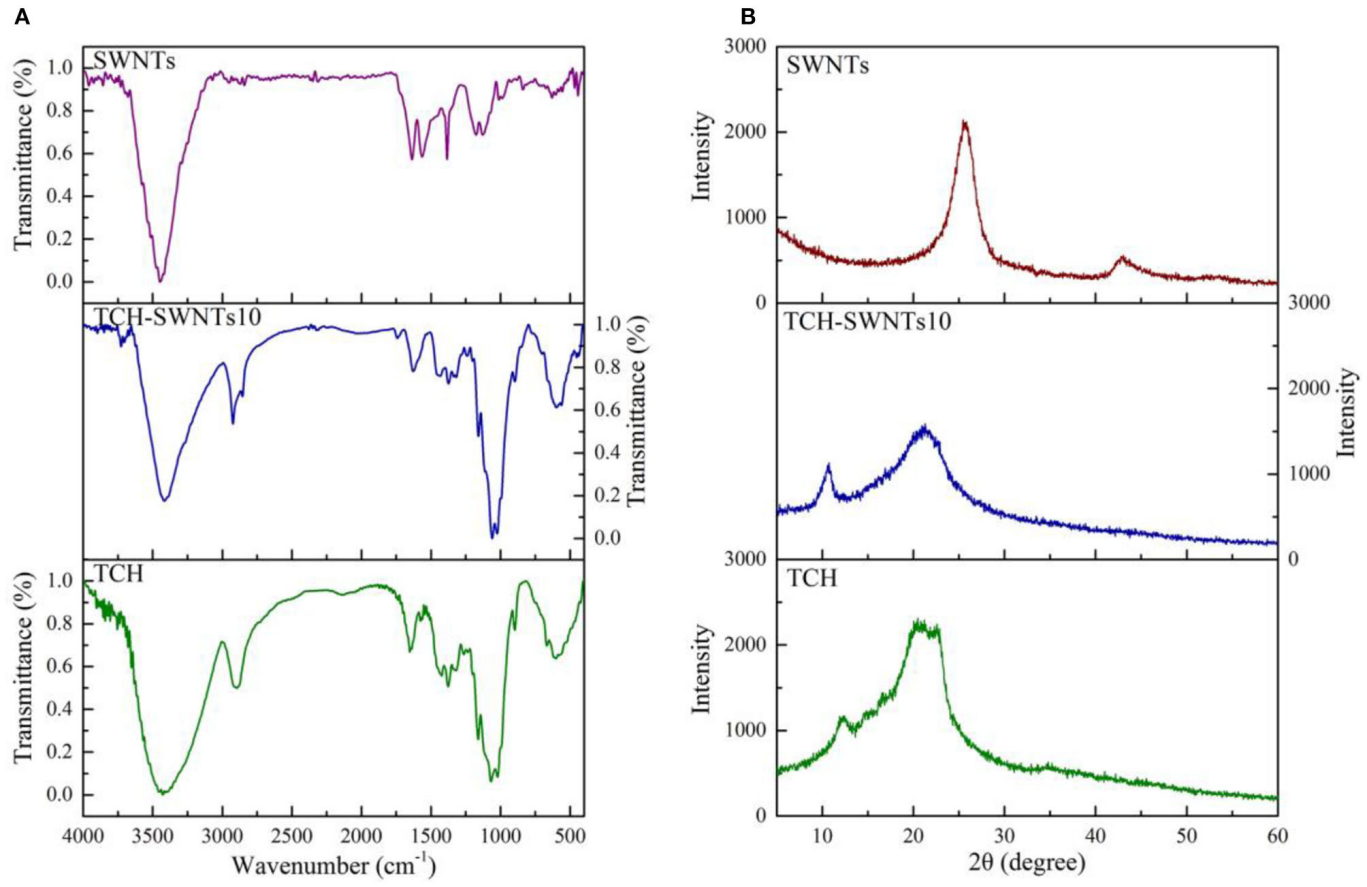
**(A)** FTIR and **(B)** XRD spectra of SWNTs, TCH-SWNTs10, and TCH.

The FTIR spectra of SWNTs, TCH, and TCH-SWNTs10 are shown in Figure 2A. For SWNTs, the characteristic peak at 3,445 cm^−1^ was assigned to the stretching vibration of the O–H groups. The characteristic peak at 1,630 cm^−1^ was due to the stretching vibration of the C–C groups in the carbon skeleton of SWNTs (Gutierrez et al., 2017). The characteristic peaks at 2,950 and 2,854 cm^−1^ were relative to the stretching vibration and deformation vibration of the C–H groups, suggesting an increase in sp^3^ carbon atom on the surface of SWNTs (Liang et al., 2004). The characteristic peaks at 1,610, 1,560, and 1,320 cm^−1^ indicated that SWNTs contain abundant oxygroups, e.g., carboxy (COOH), hydroxy (O–H), and carbonyl (C=O) (Gutierrez et al., 2017). For TCH, the characteristic peaks at 2,919, and 3,411 cm^−1^ were assigned to stretching vibration of the C–H groups in the methyl in the cellulose and the stretching vibration of O–H groups in the cellulose molecule chain (Peng et al., 2009). The intense peaks at 1,049 and 1,161 cm^−1^ were assigned to the stretching vibrations of the C–C and C–O groups (Kačuráková et al., 1994; Sun et al., 2002). The vibration of the β-glycosides bond was at 892 cm^−1^. For TCH-SWNTs10, the FTIR spectra are similar to those of TCH, but the characteristic peaks at 2,950 and 2,854 cm^−1^ were clearly enhanced due to the vibration of the C–H groups on SWNTs.

The XRD patterns of SWNTs, TCH, and TCH-SWNTs10 are shown in Figure 2B. For SWNTs, an intensive diffraction peak at about 2θ = 25.5° and a weak diffraction peak at about 2θ = 43° suggested that SWNTs were composed of a six-member carbon ring structure (Han and Zettl, 2003; Liu et al., 2012). For TCH, a broad diffraction peak at about 2θ = 21° and a narrowed diffraction peak at about 2θ = 12° suggested the crystal structure of cellulose I (Chen et al., 2011). For TCH-SWNTs10, the diffraction peaks of SWNTs were hardly observed, and the diffraction peak of TCH at 2θ = 21.0° was decreased, suggesting that the composite hydrogels with SWNTs became more amorphous than TCH. The decrease in crystallinity of TCH-SWNTs10 may be due to the higher non-uniform content in cellulose chains caused by the insertion of SWNTs (Vasconcellos et al., 2011).

### TGA Analysis

**Figure 3:**
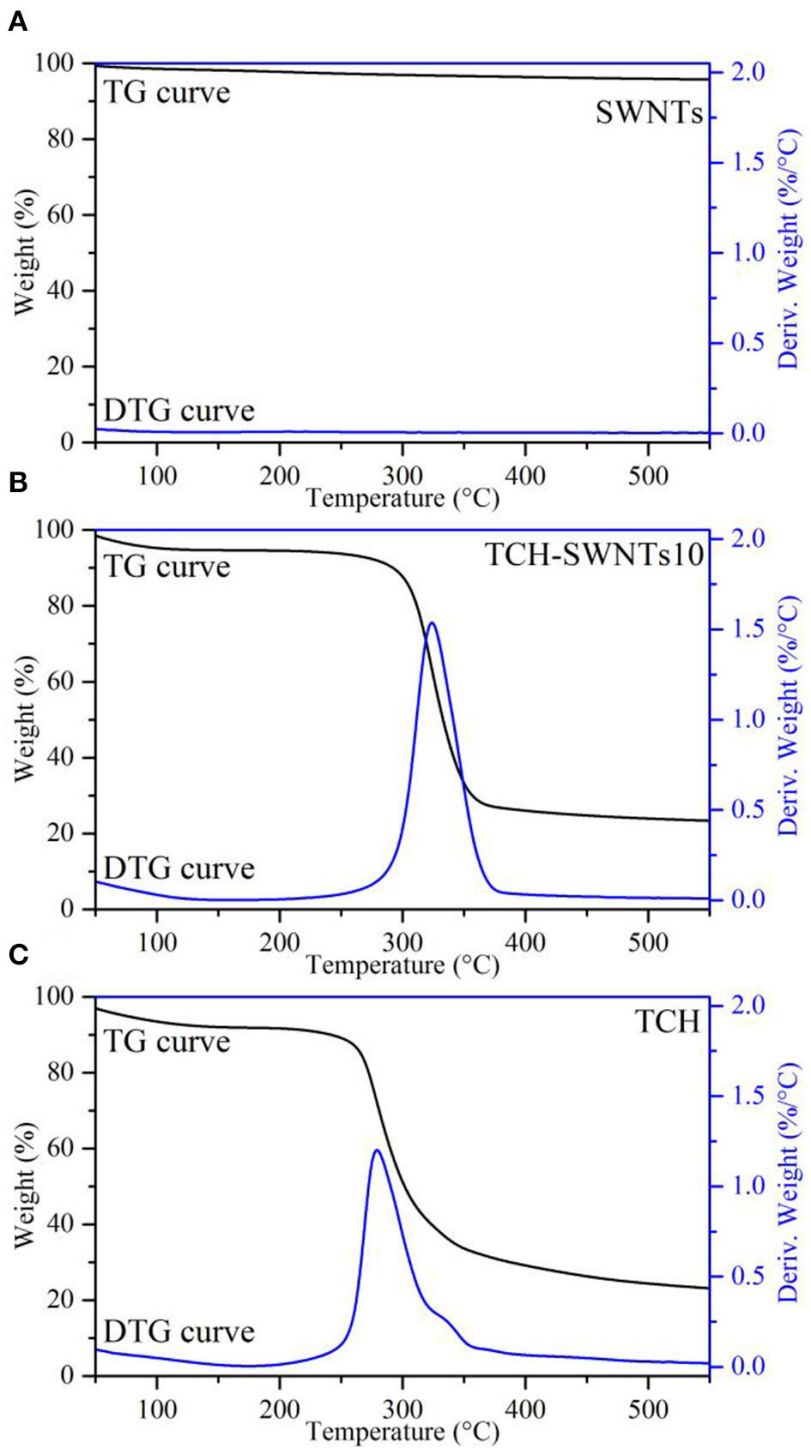
TG and DTG curves of **(A)** SWNTs, **(B)** TCH-SWNTs10, and **(C)** TCH.

The TGA and differential thermogravimetric (DTG) curves of SWNTs, TCH, and TCH-SWNTs10 are shown in Figure 3. The TGA curves show the accumulation of weight loss in the sample, whereas the DTG curves are first-order derivatives of the TGA curves and describe the changes in the temperature-dependent quality of the samples (El-Sayed et al., 2011).

As observed in Figure 3A, SWNTs showed good resistance to thermal shock. Due to water evaporation, the weight of SWNTs decreased by only 4.4% until the highest temperature of 600°C of the experiment was reached, suggesting that SWNTs are a kind of heat-resistant material (Janas and Koziol, 2013). Two weight loss steps were obviously observed from the beginning to 100°C and from 250 to 400°C in the TGA curve of TCH, as shown in Figure 3C. Because of water evaporation, 8% of the weight was lost in the first step, whereas 66% of the weight was lost in the second step due to thermal decomposition of the sample. As shown in the DTG curve of TCH, a sharp peak was observed at around 280°C accompanied by 1.2%/°C of derivative weight loss (deriv. weight). The TGA curve of TCH-SWNTs in Figure 3B was similar to that of TCH. Weight loss of 5 and 67% was observed in the first and second steps, respectively. The DTG curve of TCH-SWNTs showed a sharp peak at about 323°C along with 1.5%/°C deriv. weight loss. The upshift in decomposition temperature, from 280 to 323°C, and increase in deriv. weight in TCH-SWNTs10, from 1.2 to 1.5%/°C, indicated that the addition of SWNTs into the TCH could improve the thermal stability of the sample.

### Texture Profiles Analysis

**Figure 4:**
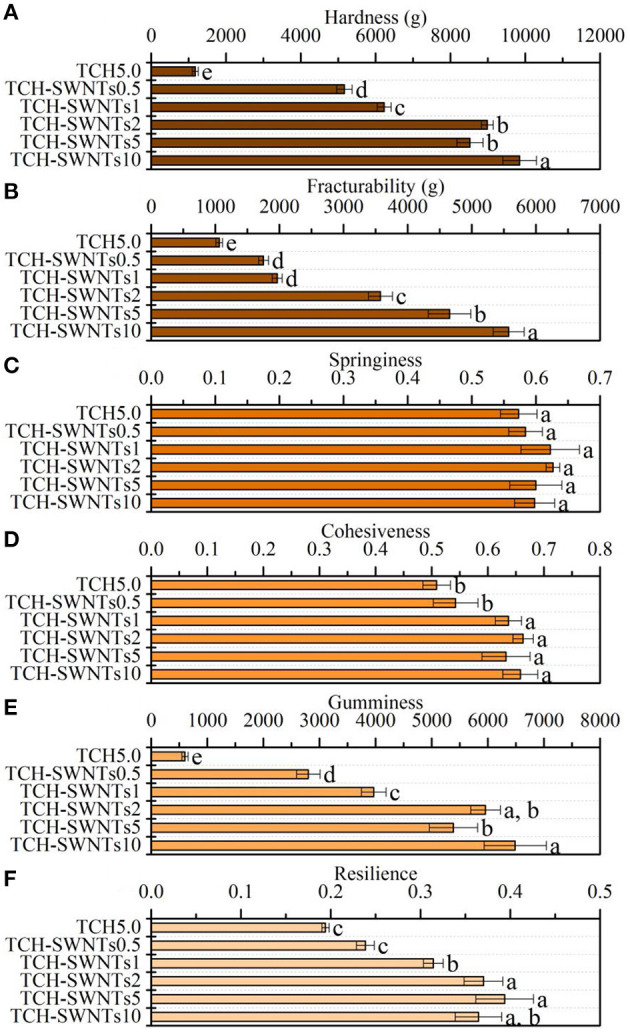
**(A)** Hardness, **(B)** fracturability, **(C)** springiness, **(D)** cohesiveness, **(E)** gumminess, and **(F)** resilience of TCH, TCH-SWNTs.5, TCH-SWNTs1, TCH-SWNTs2, TCH-SWNTs5, and TCH-SWNTs10. Each value was expressed as mean ± standard deviation. Bars marked with different letters represented significant differences between the values (*p* < 0.05).

The texture profiles of the hydrogels prepared from tea cellulose with different contents of SWNTs are shown in Figure 4. With the increase in the amount of SWNTs, the prepared hydrogels exhibited different degrees of differences in cohesiveness, fracturability, gumminess, hardness, resilience, and springiness. It was observed that TCH-SWNTs had remarkably greater hardness (Figure 4A), fracturability (Figure 4B), gumminess (Figure 4E), and resilience (Figure 4F) than the undoped TCH, i.e., the texture parameters were further enhanced with an increase in the amount of SWNTs. As an extreme instance, the hardness, fracturability, gumminess, and resilience values of TCH-SWNTs10 were 8.4, 5.3, 10.8, and 1.9 times, respectively, higher than those of TCH. Nevertheless, the springiness (Figure 4C) and cohesiveness (Figure 4D) of the hydrogels showed only a slight difference with the addition of SWNTs.

These changes in texture profile parameters may be due to the hydrogen bonds between the hydroxy groups on the cellulose chain and oxygen-containing groups on the skeleton of SWNTs, e.g., –COOH and –OH, which improved the interfacial adhesion of the composite hydrogels. Besides, SWNTs ran through the tea cellulose hydrogel network irregularly, dispersed stress transmission, and reinforced energy dissipation.

### Methylene Blue Adsorption

**Figure 5:**
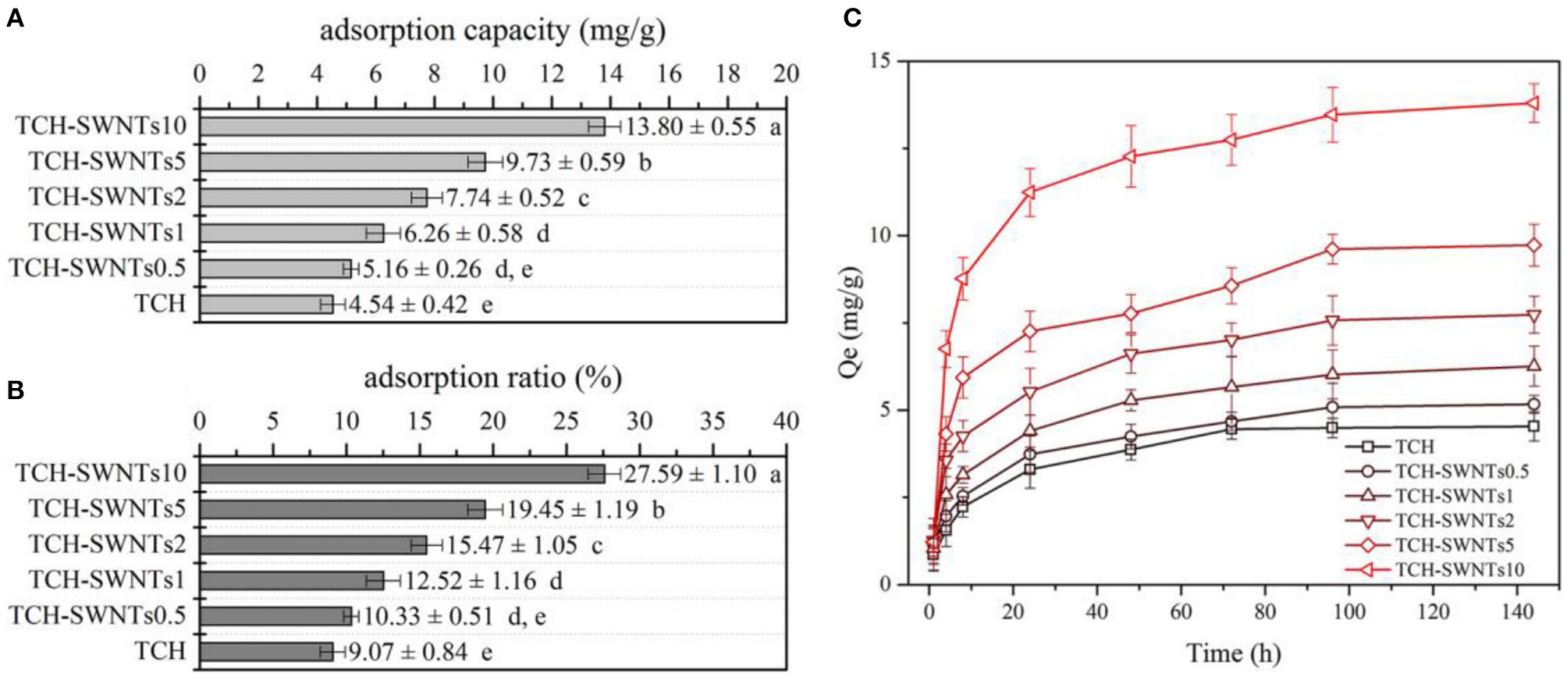
**(A)** Adsorption capacity, **(B)** adsorption ratio, and **(C)** adsorption kinetics curves of TCH, TCH-SWNTs.5, TCH-SWNTs1, TCH-SWNTs2, TCH-SWNTs5, and TCH-SWNTs10. Each value was expressed as mean ± standard deviation. Bars marked with different letters presented significant differences between the values (*p* < 0.05).

As a heterocyclic aromatic chemical compound, methylene blue can be used as medication and dyestuff, and it tends to attract negative particles (Jin et al., 2008). Figure 5 shows the adsorption capacity, adsorption ratio, and adsorption kinetics curves of the prepared composite hydrogels. It was obviously observed that TCH-SWNTs had greater adsorption capacity than TCH (Figure 5A). With the increase in the amount of SWNTs, the adsorption capacity of TCH-SWNTs improved remarkably, e.g., the adsorption capacity of TCH-SWNTs10 was about three times higher than that of TCH. The changes in the adsorption ratio of the prepared composite hydrogels were similar to those in the adsorption ratio (Figure 5B). After immersion in the methylene blue solution for 6 days (144 h) to achieve adsorption balance, TCH exhibited a maximum adsorption ratio of 9.07%, whereas TCH-SWNTs10, TCH-SWNTs5, TCH-SWNTs2, TCH-SWNTs1, and TCH-SWNTs.5 exhibited maximum adsorption ratios of 27.59, 19.45, 15.47, 12.52, and 10.33%, respectively. The adsorption capacities of TCH-SWNTs10, TCH-SWNTs5, TCH-SWNTs2, TCH-SWNTs1, TCH-SWNTs.5, and TCH were calculated to be 13.8, 9.73, 7.74, 6.26, 5.16, and 4.5 mg/g, respectively. The improvement in adsorption capacity and adsorption ratio may be due to a large number of anionic groups, e.g., –COO^−^ and –O^−^, in the skeleton of SWNTs. Moreover, it was observed from the adsorption kinetics curves (Figure 5C) that in the first 24 h, the adsorption capacity growth was rapid but then tended to flatten.

Generally, the adsorption process consisted of two processes, i.e., mass transfer and mass adsorption, and could be described and explained by the pseudo-first-order model or the pseudo-second-order model (Ho and McKay, 1999; Azizian, 2004; Foo and Hameed, 2010).

The pseudo-first-order model assuming a change in adsorption capacity with respect to time is a first-order relationship, and its integral equation is expressed as

(3) $$\begin{array}{l} \text{ln}\left(Q_{c}-Q_{t}\right)=\text{ln}\left(Q_{c}-k_{1}t\right) \end{array}$$

where *t* is adsorption time (h), *Q*_*e*_ is equilibrium adsorption capacity (mg/g), *Q*_*t*_ is adsorption capacity at a given time (mg/g), and *k*_1_ is the rate constant of apparent adsorption of the pseudo-first-order model (h^−1^).

The pseudo-second-order model assuming a change in adsorption capacity with respect to time is a first-order relationship, and its integral equation is expressed as

(4) $$\begin{array}{l} \frac{t}{Q_{t}}=\frac{1}{k_{2}Q_{e}^{2}}+\frac{t}{Q_{e}} \end{array}$$

where *t* is adsorption time (h), *Q*_*e*_ is equilibrium adsorption capacity (mg/g), *Q*_*t*_ is adsorption capacity at a given time (mg/g), and *k*_2_ is the rate constant of apparent adsorption of the pseudo-second-order model [g/(mg·h)].

**Figure 6:**
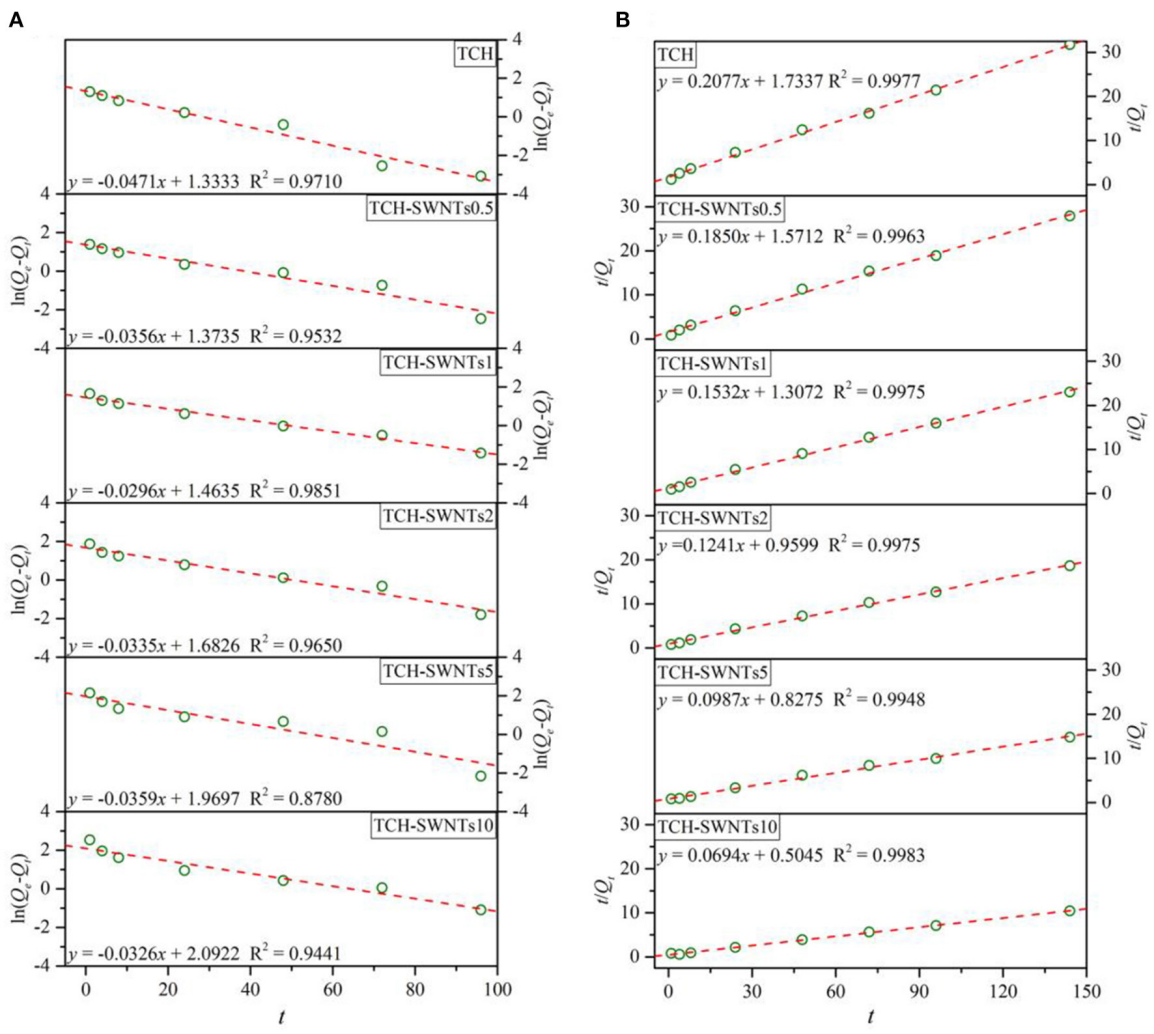
Absorption models for the absorption of methylene blue for **(A)** the pseudo-first-order model and **(B)** the pseudo-second-order model.

Figure 6 shows the linear plot of ln(*Q*_*e*_–*Q*_*t*_) vs. *t* for the pseudo-first-order model and *t*/*Q*_*t*_ vs. *t* for the pseudo-second-order model for absorption of methylene blue in the prepared hydrogels.

It is observed from Figure 6 that the experimental absorption data of all prepared hydrogels fitted well with the pseudo-second-order model. The values of the determination coefficient, i.e., *R*^2^, of the pseudo-second-order model of TCH, TCH-SWNTs0.5, TCH-SWNTs1, TCH-SWNTs2, TCH-SWNTs5, and TCH-SWNTs10 were 0.9977, 0.9963, 0.9975, 0.9975, 0.9948, and 0.9983, respectively, which were higher than those of the pseudo-first-order model. Furthermore, with the increase in the amount of SWNTs, the rate constants of apparent adsorption of the pseudo-second-order model exhibited a declining trend, so the *k*_2_ values of TCH, TCH-SWNTs.5, TCH-SWNTs1, TCH-SWNTs2, TCH-SWNTs5, and TCH-SWNTs10 were calculated to be 0.0249, 0.0218, 0.0179, 0.0161, 0.0118, and 1.0095 g/(mg·h), respectively. Besides, the values of predicted adsorption capacity, i.e., *Q*_*e*_, of the pseudo-second-order model were 4.82, 5.41, 6.53, 8.06, 10.14, and 14.41 mg/g, which were closer to experimental values (Figure 5A).

According to the three reasons mentioned above, the kinetic adsorption behaviors of the prepared hydrogels could be described and evaluated by the pseudo-second-order model, which suggested that chemical adsorption dominated the adsorption process of TCH-SWNTs.

## Conclusion

Characterization analysis of XRD and FTIR suggested successful preparation of hydrogels from tea residue cellulose with the addition of SWNTs in 1-allyl-3-methylimidazolium chloride. Observed from the results of TGA and TPA analysis, the thermal stability and textural properties of the composite hydrogels improved with an increase in the amount of SWNTs. The composite hydrogels exhibited a chemisorption behavior that was well-described by the pseudo-second-order model.

## Data Availability Statement

The raw data supporting the conclusions of this article will be made available by the authors, without undue reservation.

## Author Contributions

ZL did the most parts of experiments, analyzed data, and wrote this paper. DL provided an idea and polished up the article.

## Conflict of Interest

The authors declare that the research was conducted in the absence of any commercial or financial relationships that could be construed as a potential conflict of interest.
